# The role of serum procalcitonin and C-reactive protein levels in predicting spontaneous bacterial peritonitis in patients with advanced liver cirrhosis

**DOI:** 10.12669/pjms.326.10995

**Published:** 2016

**Authors:** Hongli Wu, Lin Chen, Yuefeng Sun, Chao Meng, Wei Hou

**Affiliations:** 1 Hongli Wu, Ph.D., Department of Clinical Laboratory Medicine, Tianjin Second People’s Hospital, Tianjin, China; 2 Lin Chen Ph.D., Department of Clinical Laboratory Medicine, Tianjin Second People’s Hospital, Tianjin, China; 3 Yuefeng Sun, Department of Clinical Laboratory Medicine, Tianjin Second People’s Hospital, Tianjin, China; 4 Chao Meng, Department of Clinical Laboratory Medicine, Tianjin Second People’s Hospital, Tianjin, China; 5 Wei Hou, Ph.D. Tianjin Institute of Hepatology, Tianjin, China

**Keywords:** Ascitic fluid infection, C-reactive protein, Procalcitonin, Spontaneous bacterial peritonitis

## Abstract

**Objective:**

To determine the role of serum procalcitonin (PCT) and C-reactive protein (CRP) in predicting spontaneous bacterial peritonitis (SBP) in patients with advanced liver cirrhosis.

**Methods:**

A total of 88 patients with advanced liver cirrhosis were enrolled for this study, which included 40 cases with SBP and 48 cases with CNNA. Bacterial cultures, ascitic fluid (AF) leukocyte, C-reactive protein (CRP) and serum PCT measurements were carried out prior to the use of antibiotics. Receiver operating characteristic (ROC) curves were used to evaluate the diagnostic performance of procalcitonin levels.

**Results:**

Serum PCT levels in advanced liver cirrhotic patients with SBP were significantly higher than those with CNNA. We used PCT 0.78 ng/mL as optimal cutoff value to diagnose SBP, for which the sensitivity and specificity was 77.5% and 60.4%. The area under the curve (AUC) was 0.706 (95% confidence interval: 0.576-0.798). The PCT level was significantly correlated with the AF WBC count (rs=0.404, P<0.01). However, there was no significant difference between SBP and CNNA patients in serum CRP levels.

**Conclusion:**

According to our findings, serum PCT levels seem to provide an early diagnostic accuracy in advanced liver cirrhotic patients with SBP.

## INTRODUCTION

Ascitic fluid infection (AFI) is a common cause of morbidity in patients with advanced liver cirrhosis. AFI, which was divided into spontaneous bacterial peritonitis (SBP) and culture-negative neutrocytic ascites (CNNA), is considered as a potential trigger factor for many complications of advanced liver cirrhosis, including hepatic encephalopathy, variceal bleeding, hepatorenal syndrome.^1^,^2^ The clinical course of SBP and CNNA was similar however, patients with SBP showed higher in-hospital mortality than those with CNNA.^1^,^3^ Early diagnosis and prompt initiation of antibiotic therapy have been considered to be crucial for the treatment of SBP.^4^ However, it is difficult to determine the early stage of SBP in the detection of bacterial infection. Ascitic fluid culture examination is time consuming (24-48h) which delays the diagnosis and treatment. Therefore, the identification of clinical and laboratory parameters for the early diagnosis of SBP would be of special interest.^5^,^6^

Over the last few years, the acute phase reactant proteins, such as C-reactive protein (CRP) and procalcitonin (PCT) have been investigated as tools for early diagnosis of SBP in various clinical conditions.^5^,^7^ It is well known that CRP and PCT increased rapidly in response to bacterial infection. They are sensitive diagnostic marker that can be used to predict diagnosis, monitor bacterial infections and guide the clinical use of antibiotics.^8^,^9^ In this context, the purpose of the study was to evaluate the performance of CRP and PCT for the early diagnosis of SBP in patients with advanced liver cirrhosis.

## METHODS

### Study population

This was a retrospective study and approved by the second people’s hospital of Tianjin. Eighty eight patients with advanced liver cirrhosis (mean age: SBP, 65.70±8.71; CNNA, 62.17±8.37; 67.5% were males) due to ascites were enrolled from the second people’s hospital of Tianjin between Jan 2012 and Jan 2015. Clinical and laboratory data were obtained from the comprehensive electronic medical record. All subjects met the following criteria: (1) liver cirrhosis with ascites and/or other complications were confirmed by medical history; (2) The ascitic fluid cultures, serum PCT and CRP measurements, ascitic fluid WBC count were performed before the use of antibiotics at admission; (3) All enrolled patients have no infection in other organs or sites; (4) The patients did not exhibit liver failure, liver cancer or fungal infection.

### Classification of patients

Ascitic fluid (AF) were collected from hospitalized patients using a sterile method and cultured in blood culture bottles, according to the guidelines of the Infectious Diseases Society of America. Bacterial identification and antimicrobial susceptibility testing were carried out according standard procedures. According to the results of microbiological test, patients were classified into two groups with respect to ascitic fluid infection including SBP (WBC count ≥ 500/mm^3^ and PMNL >250/mm^3^ in AF with a positive bacterial culture) and CNNA (WBC count ≥ 500/mm^3^ and PMNL >250/mm^3^ in AF but the culture is negative).^6^ Etiology of cirrhosis was recorded in advanced liver cirrhosis patients with respect to HBV, HCV, alcoholic and autoimmune cirrhosis according to the Child-Pugh criteria classification.^5^

**Fig.1:**
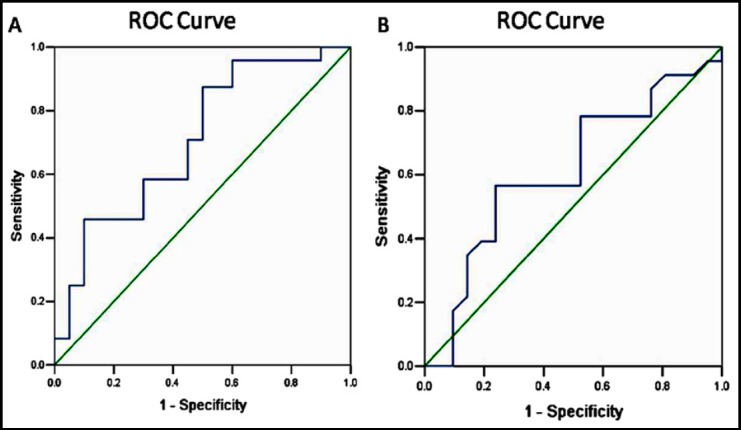
ROC curve showing the diagnostic values of PCT and CRP for advanced Liver cirrhosis patients with SBP. A: ROC curves of PCT for predicting advanced Liver cirrhosis patients with SBP; B: ROC curves of CRP for predicting advanced Liver cirrhosis patients with SBP.

### Measurement of PCT, CRP and AF WBC count

The bloods of samples were prepared according to previous study.^6^ All patients had received the measurement of PCT, CRP and AF WBC count. PCT measurement was performed using electrochemiluminescence immunoassay (ECLIA) method with Cobas immunoassay analyser (Germany). CRP quantification was performed using immunoturbidimetric assay with LIFOTRONIC (China). AF WBC count was determined using Sysmex xt -4000i (Japan).

### Statistical analysis

For variables with normal distribution, the data was reported as mean and standard deviation. Variables without normal distribution were expressed as median (minimum-maximum). Categorical variables were compared with the χ^2^-test, and continuous variables were compared with Student’s *t*-test for parametric data or a Mann-Whitney test for medians of nonparametric data. The spearman’s correlation coefficient (rs) between PCT and AF WBC count was calculated. The diagnostic performance of PCT was evaluated using a receiver operating characteristic curve. A 2-sided probability value of <0.05 was considered statistically significant. Data were analyzed using SPSS 16.0 (SPSS Inc, Chicago, IL, USA).

## RESULT

### Patient demographics

A total of 88 patients (40 SBP and 48 CNNA) with advanced liver cirrhosis had been identified with ascitic fluid infection. There were no significant differences in age, gender, Etiology of cirrhosis, Child-pugh classification between the two groups. However, patients with SBP had significantly more complications (≥2) and were easy to experience fever, abdominal pain, and rebound than patients with CNNA (P<0.05, Table-I). Furthermore, patients with SBP showed significantly higher in-hospital mortality than those with CNNA (15.0% vs 4.1%; P<0.05, Table-I). In our culture positive patients (n=40), Escherichia Coli was responsible microorganism in 18 patients (45.0%), Klebsiella pneumoniae in 11 patients (27.5%), Staphylococcus in 7 patients (17.5%) while enterococci in 4 patients (10%).

**Table-I:** Characteristics of patients with advanced cirrhosis.

		SBP (N=40)	CNNA (N=48)	P value
Age (M±SD)		65.70±8.71	62.17±8.37	p=0.948
Gender (%)	Male	24(60)	30(70.0)	P=0.236
	Female	16(40)	18(30.0)	
Etiology of cirrhosis (%)	Hepatitis B	15(37.5)	21(43.7)	p= 0.303
	Hepatitis C	13(32.5)	21(43.7)	p= 0.303
	Alcohol	8(20)	8(16.7)	
	Autoimmune	4(10)	5(10.4)	
Child-pugh classification (%)	A	0 (0)	0(0)	p=0.440
	B	8(20)	13(27.1)	
	C	32 (80)	35(72.9)	
Number of complications (%)	≥2	25(62.5)	20(41.6)	P<0.05
	<2	15(37.5)	28(58.4)	
In-hospital mortality (%)		6(15.0%)	2(4.1%)	P<0.05
Clinical Symptom (%)				
Fever		26 (65)	23 (48)	P<0.05
Abdominal pain		20(50)	31 (64.5)	P<0.05
Abdominal tenderness		15(37.5)	20 (41.6)	P=0.606
Rebound		4(10)	0(0)	P<0.05
Altered mental status		20 (50)	28 (58.3)	P=0.737

*Note:* Complications included esophageal variceal bleeding, hepatic encephalopathy or hepatorenal syndrome.

### Comparisons of PCT, CRP, and AF WBC between two groups

The results of comparisons between two groups showed that the median values of PCT, CRP and AF WBC in SBP group were 2.95 ng/ml, 30.50 mg/dl, and 2727.5 mm^3^ while 1.15 ng/ml, 34.0mg/dl, and 1161.5 mm^3^ in CNNA group, respectively. There was significant difference between the SBP and CNNA group in PCT and AF WBC count (P<0.05, Table-II). However, there was no significant difference between SBP and CNNA patients in serum CRP levels (P =0.933, Table-II).

**Table-II:** The values of median, minimum and maximum of PCT, CRP and AF WBC.

Group	PCT(ng/ml)	CRP(mg/dl)	AF WBC(mm^3^)
SBP (n=40)	2.95(0.29-27.04)	30.50(5-150)	2727.5(546-31312)
CNNA (n=48)	1.15(0.14-15.24)	34.0(5-150)	1161.5(521-7074)
	P=0.003	P =0.933	P =0.015

### Correlations between AF WBC count and PCT

We also performed correlations between AF WBC count and PCT and observed a significant positive correlation between PCT and AF WBC count (rs=0.404, *P*<0.01).

### Diagnostic accuracy of PCT

The ROC analysis results revealed that the optimal cutoff value for PCT was 0.78ng/ml with sensitivity 77.5%, specificity 60.4% (AUC: 0.706, CI 95%: 0.576-0.798, p<0.01). Meanwhile, the optimal cutoff value for CRP was 15.53 ng/ml. The sensitivity and specificity of CRP for liver cirrhosis patients with SBP were 75% and 61.2%, respectively (AUC: 0.613, CI 95%: 0.532-0.724, p<0.01).

## DISCUSSION

Patients with advanced Liver cirrhosis are very susceptible to bacterial infections because of acquired immune defects and bacterial translocation, which increases ascitic fluid infection. SBP and CNNA are regarded as serious complications of advanced liver cirrhosis.^10^ Runyon BA reports showed there was no significant different in mortality between SBP (32 patients) and CNNA (17 patients).^11^ On the contrary, the study of Kim SU in 2010, 37 patients with SBP and 93 patients with CNNA, showed patients with SBP represented a significant higher in-hospital mortality than CNNA (16.2 vs 4.3%; P = 0.031) which was consistent with ours.^1^ In our study in-hospital mortality in patients with SBP is also significant higher than in CNNA (15.0% vs 4.1%, P<0.05). Furthermore, SBP patients were more frequently to have complications (≥2) than CNNA patients which possibly indicated that CNNA is a less severe variant of SBP.

SBP is a severe complication of advanced cirrhosis. Abdominal pain and fever are the most common symptoms however, symptoms sometimes may be subtle, accidentally, and entirely without any clinical manifestation. Escherichia coli, Klebsiella pneumoniae and other Enterobacteriaceae have been reported as the species most frequently cause SBP via bacterial translocation.^11^-^13^ Accordingly, in our study, infections with the predominance of Gram negative over Gram positive bacteria, Escherichia Coli was responsible microorganism in 18 (45.0%) patients followed by Klebsiella pneumoniae in 11 patients (27.5%), Staphylococcus in 7 patients (17.5%) while enterococci in 4 patients (10%). Though Ascites fluid culture is the current gold standard for detection of AFI, the practical value of ascites culture in diagnosis of SBP is limited because performing ascites culture is time consuming.^14^-^16^ Therefore, diagnostic paracentesis with high sensitivity and specificity for the diagnosis of SBP are needed.

PCT is a peptide that is composed of 116 amino acids and synthesized by C-cells of thyroid gland. The normal serum concentration of PCT was always < 0.05 ng/ml. The level of PCT will increase rapidly after bacterial infections and the effect can be detected even 2-4 hours after inflammatory response. It was reported that the PCT level was significantly higher in SBP than CNNA.^6^,^16^,^17^ Cekin et al. demonstrated that serum PCT levels may be used as a marker for the diagnosis of SBP in patients with cirrhosis via comparing 20 SBP patients and 39 CNNA patients.^6^ In this study, we found that PCT had a higher AUROC for predicting SBP. Furthermore, we identified a cutoff value (0.78 ng/mL) for PCT to rule out the presence of SBP. The AUCROC for PCT was 0.706 and PCT cutoff value was 0.78 ng/mL (sensitivity 77.5%, specificity 60.4%) while for CRP were 0.613 and 15.53 ng/ml (sensitivity 75.0%, specificity 61.2%), respectively. Furthermore, our results revealed that PCT concentration was correlated with AF WBC, so serum PCT levels should be considered in combination with AF WBC for the early diagnosis of SBP.

CRP is a well-known but nonspecific systemic inflammatory biomarker which is synthesized in the acute phase of inflammation.^18^ It also rises obviously in the case of trauma, burns, myocardial infarction and cancer.^19^,^20^ Cekin et al. studied 101 patients and found serum PCT may be a satisfactory diagnostic marker than CRP in patients with liver cirrhosis related ascites.^6^ The basic level of CRP in patients with cirrhosis is higher than in patients without cirrhosis, but when infection occurs the more serious the potential liver dysfunction, the lower the increase in CRP.^21^ Therefore, the predictive power of CRP for infection is weak in patients with advanced cirrhosis.^22^ Simultaneously, our findings revealed that there was no significant difference between SBP patients and CNNA patients in serum CRP, which suggested that CRP in AFI patients seems not to provide satisfactory diagnostic accuracy. Serum PCT levels appear to have many advantages over the traditional methods in early diagnosis of SBP in advanced Liver cirrhosis, such as within two hour rapid detection, the nonintrusive diagnostic test, and bedside availability.^17^ Therefore, PCT levels could serve as a simple utility for confirmation or exclusion of SBP in advanced liver cirrhosis patients.

In conclusion, PCT level was higher in advanced Liver cirrhosis patients with SBP than CNNA which indicated it may represent as a simple biomarker for differentiating SBP from CNNA. PCT may be a prognostic predictor to guide the empirical antimicrobial therapy in order to decrease the in-hospital mortality and the frequency of complications.
